# Understanding Patient Perceptions of Bacterial Vaginosis Treatments: Mixed Methods Sentiment Analysis Study of Online Drug Review Forums

**DOI:** 10.2196/71720

**Published:** 2025-10-10

**Authors:** Eren Watkins, Andrea N Cimino, Curtis Culbertson, Juliana Raymaker, Jennifer R Amico

**Affiliations:** 1 Medical Affairs & Outcomes Research Organon & Co Jersey City, NJ United States; 2 Rogue Scholar Consulting Baltimore, MD United States; 3 Family Medicine and Community Health Robert Wood Johnson Medical School Rutgers Health New Brunswick, NJ United States

**Keywords:** bacterial vaginosis, bacterial vaginosis symptoms, patient experiences, bacterial vaginosis treatment, sentiment analysis, natural language processing, qualitative

## Abstract

**Background:**

Bacterial vaginosis (BV) is the most common cause of vaginal discharge in people of childbearing age in the United States. More information about what patients do and do not like about the most common BV products and the extent to which they reduce BV symptoms is important for understanding patients’ health and the current treatment landscape for BV.

**Objective:**

Using data from online drug review forums, this study’s objectives were to (1) quantitatively characterize the patient voice via sentiments (positive to negative) and emotions about the three most common Food and Drug Administration (FDA)–approved treatments for BV—oral metronidazole (OM), vaginal metronidazole (VM), vaginal clindamycin (VC)—and (2) qualitatively summarize themes characterizing the patient-perceived impact of BV and BV products.

**Methods:**

Data for this mixed methods descriptive study came from 1645 users’ reviews of BV products posted on WebMD.com and Drugs.com. Reviewer attributes, reviewer-submitted star ratings, and sentiment analysis (SA) using word-emotion association were analyzed with descriptive statistics and bivariate associations. A traditional qualitative analysis using qualitative description was also performed.

**Results:**

Most reviewers were female (n=629, 99.4%), between the ages of 18 and 44 years, and reported using BV products for less than 1 month, though qualitative results suggested most reported recurrent BV infections. Quantitative results revealed reviewers’ preference for vaginal products. The mean star ratings for VC were significantly higher when compared to OM and VM. VC reviews had the highest proportion of positive emotion words compared to OM and VM. Qualitative results for VC supported the quantitative findings: favorable themes related to perceptions of value, effectiveness in alleviating symptoms, and minimal side effects. Additionally, despite some concerns related to the cost of VC, reviewers said they would use the medication again. Other qualitative findings supported BV medical education campaigns for patients and providers on BV treatment.

**Conclusions:**

Overall, people want a BV treatment that is easy to use, quickly alleviates symptoms, and has minimal side effects. Patients use product reviews to inform their decision-making about BV treatment, ask and seek answers to health-related questions, and share their experiences, presenting a unique opportunity for comprehensive patient education through clinical encounters or public health outreach efforts.

## Introduction

### Background

Bacterial vaginosis (BV) is the most common cause of vaginal discharge in people of childbearing age in the United States [1], with approximately one-third (29%) of those aged 14-49 years having experienced either symptomatic or asymptomatic BV [2]. BV is caused by an overgrowth of anaerobic bacteria naturally found in the vagina, causing symptoms such as itching, a “fishy” odor, burning while urinating, and changes in the color and consistency of vaginal discharge [2]. BV acquisition is associated with myriad factors, including sexual health and hygiene practices (eg, vaginal douching, having multiple male or female sexual partners) [3,4], stress exposure, smoking, and being African American [4-6]. BV is associated with increased susceptibility to sexually transmitted infections (STIs), including HIV [7]; gynecological conditions, such as pelvic inflammatory disease (PID) and endometritis [3]; and adverse pregnancy outcomes, such as pregnancy loss and miscarriage, preterm labor, and low birth weight [3,8,9].

Several prescription medications (eg, nitroimidazole, clindamycin) and over-the-counter (OTC) products, such as boric acid, are commonly used to treat BV [10]. Treatment selection is often made based on availability, side effects, and cost [11]. Many health care providers who are satisfied with the status quo have developed the practice of routinely choosing from among a few “tried and true” (eg, oral metronidazole [OM]) [12] treatments that are easily available, inexpensive, and familiar. For example, in a recently published study to estimate the frequency of BV recurrence and treatment patterns among 140,826 commercially insured patients, the most commonly prescribed BV medication was OM (73.6%) [13]. Although BV treatments result in 80% and 90% cure rates [14], more than 50% of women experience BV reinfection within months of initial treatment [15,16].

The internet is a key information source for patients with BV infection [17], particularly given the stigma and negative impact on the quality of life associated with BV and its symptoms [18,19]. Online reviews of BV products can be a powerful tool patients use to evaluate perceived effectiveness, side effects, and other information that may ultimately influence their decision to use a product. These unsolicited, unfiltered product reviews are a novel data source to support real-world evidence generation of the patient voice. Even Food and Drug Administration (FDA) [20] guidance recognizes the potential of online tools as useful data sources “to complement literature review findings, inform the development of research tools (e.g., qualitative study discussion guides), or as a supplement to traditional research approaches (e.g., literature, one-on-one interviews, focus groups, or expert opinion).” Understanding what people do and do not like about these products and the extent to which a product reduces their BV symptoms is an important step to understanding patients’ health and the current treatment landscape for BV.

### Sentiment Analysis to Uncover Opinions

Unsolicited, unfiltered product reviews are a novel data source to support real-world evidence generation of the patient voice. However, mining online product reviews yields enormous amounts and types (structured vs unstructured) of passively collected data that are challenging to analyze. Sentiment analysis (SA), otherwise known as opinion mining, is a type of natural language processing that calculates the degree of positive, negative, or neutral sentiments based on words or phrases in a text [21]. SA uses various algorithms to assign a numerical score to precompiled lexicons or dictionaries of words or phrases previously coded in linguistic research. For example, the *Word-Emotion Association Lexicon* created by Mohammad and Turney [22] at the National Research Council Canada (NRC) contains 13,889 words and has been used to analyze sentiment and emotions in words, sentences, tweets, and entire documents from a variety of fields, including psychology, behavioral and social sciences, and psycholinguistics. SA algorithms can be complex, leveraging sentence structure or clauses with contrasting sentiments to assign weights, or they can be straightforward counts of positive and negative words referenced in a lexicon. The benefit of using SA is that it can quickly categorize reviewer sentiment about a product and provides a common metric to compare sentiments across products.

The purpose of this study was to gain a better understanding of patient-reported sentiment toward BV medications from user reviews of BV products identified via online (public) drug review forums. The study focused on three FDA guideline–recommended BV treatments: OM, vaginal metronidazole (VM), and vaginal clindamycin (VC). The objectives of the study were to (1) quantitatively characterize patient-reported star ratings, sentiments (polarity of positive to negative), and emotions about BV and BV products from patients who had submitted product reviews and (2) qualitatively summarize themes characterizing the patient-reported impact of each BV product on the quality of life and health care provider interactions.

## Methods

### Study Design

This mixed methods, descriptive study examined BV product reviews from Drugs.com and WebMD.com for OM, VM, and VC. We selected these websites because of their ubiquitous popularity. WebMD.com is “the best known and most popular consumer-oriented health site” [23], whereas Drugs.com “has more than 50 million users each month, most of whom are in the US” and provides its users with “trusted, accurate, and ethical information about thousands of medications” [24].

### Ethical Considerations

The study protocol was approved by Solutions IRB (protocol #2022/12/19), an independent institutional review board service, as nonhuman subject research because the data were publicly available for secondary analysis, included no identifiable information, and involved no interaction between investigators and participants. An additional review from the study’s sponsor included timely reporting of pharmacovigilance and resolution of ethical concerns.

### Sample of Extracted Reviews

A custom web scraper was developed to automate downloading and organizing of the data into a relational Structured Query Language (SQL) database for analysis in R (R Foundation for Statistical Computing). Data included reviews for OM, VM, and VC used for “bacterial vaginosis” or “vaginosis caused by bacteria,” as selected by the reviewer upon posting a review. Eligible reviews were (1) in English, (2) from the United States, and (3) beginning with the earliest review available from September 25, 2007, through September 28, 2023. Reviews were ineligible if the reviewer used the product for a non-BV condition (ie, yeast infection, urinary tract infection, *Gardnerella* infection, trichomoniasis). A SQL query aided in identifying these ineligible reviews, which were manually verified before excluding. Of 1733 reviews that were scraped, 88 (5.1%) were ineligible, leaving a total sample of 1645 (94.9%) reviews.

### Procedure

#### Data Cleaning

Extracted reviews were first examined to determine whether they contained reportable adverse events (AEs). No reportable AEs were detected. Next, we examined the authenticity of the reviews to ensure a bot was not repeatedly posting the same review. Thus, we deployed a similarity analysis to look for reviews with >5 words that matched another extracted review by 90% or more. One pair of reviews for OM showed the same reviewer posted a mostly similar review to both WebMD.com and Drugs.com; therefore, these reviews were deemed real and were not excluded from the extracted sample. Next, we followed industry-standard steps [22] to prepare the data for SA, including (1) data cleaning (ie, converting text to lowercase, removing/replacing special characters, translating emoticons into their Unicode names/descriptions, expanding contractions, and correcting typos), (2) word-level tokenization (ie, splitting up into machine-readable units), and (3) word-level standardization (“lemmatization”) to reduce a word into its root or base form. Original and transformed reviews were retained in the SQL database.

SA was conducted using the NRC’s *Word-Emotion Association Lexicon* [22]. In addition to positive and negative sentiments, this first-of-its-kind lexicon further categorizes words to one or more of eight universal emotions based on Plutchik’s Wheel of Emotions [25,26]: anger, fear, anticipation, trust, surprise, sadness, joy, and disgust. The wheel offers a structured and nuanced way to visualize emotions and their variation in intensity (eg, low anger is annoyance, high anger is rage) and in combinations (eg, joy + trust = love). The NRC uses a count of positive words and negative words, which can be summed to derive a single score or presented as a proportion by dividing the sum by the total number of words in a corpus, such as all reviews for a product. Words can be associated with multiple emotions. For instance, the NRC lexicon classifies the word “smell” as negative and links it to both anger and disgust emotions.

#### Narrative Topic Classification

Individual reviews typically contained multiple topics—for instance, how BV affected the patient’s health and relationships, side effects of products tried in the past, and the value of the reviewed product—which introduced random variability, or noise, in the SA scores. For instance, a reviewer who gave a 5-star review may paradoxically have a negative sentiment score if they discuss a life-long struggle with BV and a poor interaction with a health care provider. Thus, it was necessary to determine the variety of topics discussed by the reviewer. These classifications generally followed nine topic areas described in Table 1.

**Table 1:** Topic classifications and definitions.

Code	Definition
Value	Positive, negative (“did not work”) and neutral valuation (“no side effects”) statements by reviewers about the product’s efficacy, price, or worth and whether they would use the product again
Side effects	Any intended or unintended negative effects of the reviewed product
Adherence	Whether reviewers stopped taking the product and why
Product characteristics	Features of the product, such as attributes, taste, consistency, method of delivery, and packaging
Recommended use	Statements about how to use the product and minimize side effects or tips to more effectively manage BV^a^ symptoms
Cross product	Statements discussing the effectiveness or experience using another FDA^b^ or OTC^c^ product to treat BV
Condition	Statements about the disease state (symptoms, triggers, recurrence, impact on life or relationships, etc)
Doctor interaction	Statements describing an interaction or conversation with a doctor, pharmacist, or other health care professional
Miscellaneous	All other statements, such as treatment plan/dosage, reviewer questions, words of encouragement, myths about BV, ways to improve the product, and other topics

^a^BV: bacterial vaginosis.

^b^FDA: Food and Drug Administration.

^c^OTC: over the counter.

The classification of topics was derived inductively through open coding of a sample of reviews by one author (AC), who then developed a codebook for deductive application to the remaining reviews [27]. Three authors (AC, EW, and SW) divided the reviews for individual coding. A second author verified all classifications to ensure consistency in coding across the dataset. A review could contain multiple classifications; however, each narrative segment (ie, a string of words) was coded with a single classification.

The topic classification informed both quantitative and qualitative analyses. Classifications related to the reviewed product’s value, side effects, and adherence challenges were most relevant to the quantitative analysis of star ratings and SA scores, while those reflecting patient struggles with BV, poor doctor interactions, or dissatisfaction with prior treatments contributed primarily to the qualitative analysis.

### Key Variables

#### Product Information

The product name, formulary, and route of administration (oral vs vaginal) were extracted from both websites (all categorical).

#### Reviewer Attributes

All reviewer attributes were categorical. WebMD.com collected the most reviewer information, including patient gender (male, female, nonbinary), age range (from 0-2 years to 75 years and over), and time on medication (from <1 month to 10 years or more). Gender was missing for 13.6% of reviews, the age range was missing for 3.4% of reviews, and time on medication was missing for 7.4% of reviews on WebMD.com. Drugs.com only collected information on time on medication (from <1 month to 10 years or more), and 45.7% of reviews were missing these data.

Star ratings were a primary dependent variable (continuous), with higher values indicating a more positive rating. The same product was reviewed on both Drugs.com and WebMD.com. Drugs.com asked a single question: “On a scale from 1 to 10, select how effective you found this medication, taking into consideration the benefits, side effects, and ease of use, from 1 (not effective) to 10 (most effective).” WebMD.com asked reviewers to rate the product on three separate attributes—overall satisfaction, effectiveness, and ease of use—on a scale ranging from 1 (very poor) to 5 (excellent). First, we standardized the scale by averaging the three items (min=0.5, max=5). Next, we examined whether there were statistically significant differences for the same product by website using independent *t* tests, and none were found. Because the scores did not differ by website, we averaged the star ratings for each product. Star rating scores were created for the entire review (“overall”) and by classification.

NRC emotion proportions were used to characterize all words for an entire product associated with eight emotions. Emotion proportions were used to compare emotions across products because a net count would bias reviews that contained more words. The following formula was used:



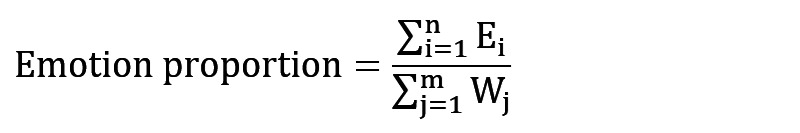



Here, 
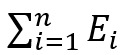
 is the sum of the occurrences of words associated with one of the eight emotions (where n is the total number of emotion-associated words), and 
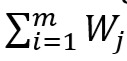
 is the sum of all words in the product (where m is the total number of words in the product text).

#### Classification Proportions

We quantified the proportion of reviews that mentioned each of the nine classifications as they represent the variety of topics mentioned for a given product. The following formula was used:



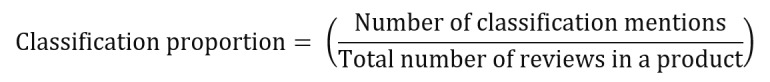



### Statistical Analysis

Quantitative data were summarized with descriptive statistics to characterize the sample of reviews including product information, reviewer attributes, and star ratings. Next, *t* tests (two-tailed, α=.05) were used to compare star ratings by reviewer attribute. For between-product comparisons, ANOVAs with Tukey correction (two-tailed, α=.05) were used with the star rating as the dependent variable. Visualizations were a key aspect of the quantitative data analysis and enabled comparisons by product. Emotion word proportions were depicted with bar charts. Classification proportions were depicted with clustered bar charts to compare the contributions of each classification category.

#### Qualitative Analytic Approach

In addition to the classification coding described before, a second qualitative analysis was performed using the 40 longest reviews for each product as these were the richest cases. Many qualitative analyses require 20-30 cases to support in-depth, iterative analysis [27,28]. A sample size of 40 was selected to achieve *information power* (rather than information redundancy or saturation), a concept that suggests that the number of participants needed increases when the sample provides less information, is less relevant to the study aims, offers less depth of cases, or requires more complex analysis [29,30]. We quantified the sample’s information power. Analysis showed that the selected cases contained multiple emotions. They had substantial overlap with reviews containing the highest-rated emotion scores; on average, the longest 40 reviews overlapped with 70% of the top emotion scores for VC (n=94), 56% for VM (n=195), and 32% for OM (n=1332). Lastly, the average word count in the 40 longest reviews was 139.6 (VC), 179.2 (VM), and 282.5 (OM) words.

The chosen analytic approach was qualitative description [31] because it offers a “rich, straight description of an experience or an event” [32] without the imposition of a theoretical or interpretative lens and aligned with the study aim to describe the reviewers’ experience in their own language. Open and inductive coding methods were used, focused on describing patterns and themes rather than interpretive results. Only one author (AC) performed the descriptive qualitative analysis; however, themes aligned with the double-coded and verified classifications, increasing trustworthiness of the findings.

## Results

### Sample Description Reviewer Attributes

Of the 1645 reviews that met the eligibility criteria, 82% (n=1349) were for OM, followed by 12.3% (n=202) for VM and 5.7% (n=94) for VC (Table 2).

Nearly two-thirds of the total sample (n=993, 60.4%) was from Drugs.com. Metronidazole comprised the largest formulary (n=1551, 94.3%: oral, n=1349, 87%; vaginal, n=202, 13%). Only 18% (n=296) of reviews were for vaginal products. A majority of Drugs.com reviewers were on the medication for less than 1 month (n=499, 94.2%). Reviewer attributes from WebMD.com showed the sample was primarily female (n=629, 99.4%), between the ages of 18 and 44 years (n=574, 81%), and took the medication for less than 1 month (n=560, 92.6%). The 4 (0.6%) nonfemale cases included 1 (25%) marked as “nonbinary” and 1 (25%) “male,” both of whom referenced having a vagina in their review; the other 2 (50%) cases did not contain enough review information to determine the sex assigned at birth.

**Table 2:** Number and breakdown of eligible reviews by product.

Product	Star rating + review text (n=1536), n (%)	Star rating + no review text (n=80), n (%)	No star rating + review text (n=100), n (%)	Total (N=1645), n (%)
VC^a^	88 (5.7)	5 (6.3)	1 (1.0)	94 (5.7)
VM^b^	189 (12.3)	8 (10.0)	5 (5.0)	202 (12.3)
OM^c^	1259 (82.0)	16 (20.0)	74 (74.0)	1349 (82.0)

^a^VC: vaginal clindamycin.

^b^VM: vaginal metronidazole.

^c^OM: oral metronidazole.

### Disease State

Results describing the disease state (qualitative n=120) from the 40 longest VC, OM, and VM reviews showed that most reviewers reported suffering from recurrent BV (VM=20, OM=11, VC=17), ranging from a few months to 14 years. A portion of reviewers reported having asymptomatic BV that was diagnosed at a routine well-woman exam. More reviewers who self-reported this was their first time with BV left reviews for VM (n=10), followed by OM (n=8) and VC (n=1). The BV symptoms reported were a “fishy” smell, itching, and irritation. BV triggers included menstruation, unprotected vaginal or oral sex, ejaculation, gynecological conditions (eg, childbirth/pregnancy), intrauterine device (IUD) insertion, hysterectomy, and, to a lesser extent, douching and superabsorbent tampons.

### Star Ratings

There were no statistically significant differences in star ratings by time on medication (Table 3), the only demographic characteristic collected by WedMD.com and Drugs.com. Products administered vaginally (mean 3.5, SD 1.48) had statistically significant higher star ratings compared to oral products (mean 3.31, SD 1.44, *t*_423.92_=–2.34, 95% CI –0.41 to –0.04, *P*<0.05). VC had the highest star rating (mean 3.85, SD 1.32), followed by VM (mean 3.39, SD 1.52) and OM (mean 3.31, SD 1.44). This difference in star ratings between the products was statistically significant (*F*_2,1562_=5.98, *P*<.01), with VC being 0.54 points higher than OM (95% CI –0.96 to –0.10, *P*<.01) and 0.46 points higher than VM (95% CI –0.88 to –0.03, *P*<.05). VM and OM were not significantly different. Finally, OM had the lowest average star rating among reviewers who made statements about side effects (mean 2.79, SD 1.46), followed by VC (mean 3.02, SD 1.53) and VM (mean 3.07, SD 1.5).

**Table 3:** Product star ratings for the review overall and by classification.

Classification^a^	VC^b^ (n=94)			OM^c^ (n=1349)			VM^c^ (n=202)	
	Star rating, mean (SD)	Classifications, n	Star rating, mean (SD)		Classifications, n	Star rating, mean (SD)		Classifications, n
Overall	3.85 (1.32)	94	3.31 (1.44)		1349	3.39 (1.52)		202
Value	4.08 (1.25)	177	3.45 (1.48)		2593	3.62 (1.05)		330
Side effects	3.02 (1.53)	73	2.79 (1.46)		2142	3.07 (1.5)		262
Adherence	0.5 (0.71)	2	1.94 (1.31)		282	1.87 (1.21)		21
Product characteristics	3.98 (0.63)	12	3.48 (1.27)		525	3.63 (1.47)		31
Recommended use	3.68 (1.21)	38	3.83 (1.19)		1233	3.73 (1.58)		79
Cross product	3.67 (1.43)	49	3.03 (1.54)		247	3.17 (1.62)		86
Condition	3.75 (1.2)	73	3.39 (1.44)		1191	3.39 (1.56)		305
Doctor interaction	3.21 (1.41)	17	2.63 (1.62)		153	2.96 (1.89)		46
Miscellaneous	3.59 (1.4)	76	3.47 (1.45)		1170	3.43 (1.54)		180

^a^The number of classifications may exceed the total number of overall reviews, as a single review could contain multiple narrative segments (ie, a string of words) assigned to a classification.

^b^VC: vaginal clindamycin.

^c^OM: oral metronidazole.

^d^VM: vaginal metronidazole.

### NRC Emotion Word Proportions

Comparing the proportion of positive and negative emotion words across products (see Multimedia Appendices 1 and 2, respectively) revealed that VC reviews had the highest proportion of three of four positive emotion words, except surprise, which was about even for VC and OM. VM and OM reviews had similar proportions of positive emotions, except for trust and surprise, which was higher for OM. In addition, OM reviews had the highest proportion of all four negative emotion words compared to VC and VM. Negative emotion word proportions for VC and VM were about the same, except for fear, which was higher for VC, and disgust, which was higher for VM.

### Classification Proportions

**Figure 1:**
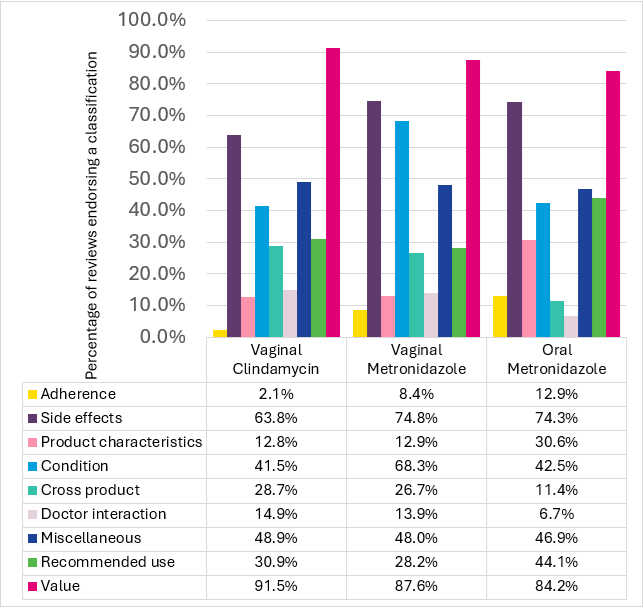
Classification proportions.

Product classifications—themes describing the variety of topics within a single review—were depicted as a proportion of reviews for a given product (Figure 1). Value statements were the highest among VC reviews (n=86, 91.5%) compared to reviews for VM (n=177, 87.6%) and OM (n=1136, 84.2%). Difficulties in adherence (yellow) were the highest in reviews for OM (n=174, 12.9%) and the lowest in reviews for VC (n=2, 2.1%). Complaints of side effects (purple) were the lowest in reviews for VC (n=60, 63.8%) compared to reviews for OM (n=1002, 74.3%) and VM (n=151, 74.8%).

### Value of the Reviewed Products

Themes on value included narrative segments about each product’s efficacy, price, and worth, as well as the user’s willingness to use the product again. Based on the 40 longest reviews for each product (n=120), regardless of the product, most reviewers were hopeful and elated to finally feel relief from BV. For example, a VC reviewer said:

I am so happy…I hope this is the end to my BV nightmare.

Another said:

This is the best I have felt in 6 months.

Similarly, an OM reviewer said:

I am so happy with the results of this medicine…I just hope I never get BV again!!!!!

A few reviewers with recurrent BV tried to moderate expectations. For example, one said:

I like [VC] better than the alternatives. Do not expect anything to be a miracle cure for this problem.

Another reviewer said that after failing multiple rounds of an oral treatment she “was told to do [VM] twice a week for 6 months,” which “kept it at bay but [I] never got rid of it.”

VC and VM reviewers reported symptom relief within 1-2 days compared to 2-3 days for OM reviewers. Positive value statements for VC included testimonies such as:

[The] next day, I felt so much better…I was dancing around the house…with not a symptom of any kind.

[I] feel so clean and better now than ever before without any side effects.

More than half of the VC users said they would take the medication again, whereas at least 4 VC reviewers said they would not recommend it because it resulted in a severe and treatment-resistant yeast infection. Only 1 VC reviewer stopped using it because of “‘wicked’ stomach pain and diarrhea” as well as “feeling like my vajayjay was on FIRE…” within minutes of inserting the VC ovule. Three VC reviewers complained that the product was expensive, even with insurance or a coupon.

Of the 40 reviews analyzed, 14 (35%) VM reviewers reported symptom relief, 15 (37.5%) said they would use VM again, 6 (15%) said they would try something else, 2 (5%) found no relief, and 2 (5%) felt it was too soon to say whether the treatment worked for them. In addition, 1 (2.5%) VM reviewer gave it a 5-star rating “simply because it worked exactly as intended and cleared up BV probably within the first two days…everything is brand new and healthy,” while 6 (15%) VM reviewers said it caused uncomfortable side effects. Those with burning and yeast infections said they would not use the medication again. Only 1 (2.5%) reviewer said she stopped using the product due to side effects, such as cramps and anxiety, but noted she “made the mistake of reading reviews” before starting treatment and was “terrified” to take VM. Three reviewers finished the treatment, however, but stated they would not take it again. One specifically disagreed with taking a vaginal medication:

I do not ever want to take this med again, and if I have to, I will insist on oral form. Why would they even make a med that had to be inserted vaginally when that area is so irritated from the infection?

Although 10 OM reviewers reported symptom relief, the vast majority of reviews contained negative value statements about its side effects—described by one as “hell on earth.” More OM reviewers said they would not use it again due to side effects compared to the other products. A few OM reviewers said the treatment did not work, as their symptoms persisted or worsened. Some reviewers commented that OM is expensive and not worth it, given its poor effectiveness and side effects. One said:

My advice…pay more out of pocket and opt for the vaginal gel as it was more effective for me and few to none with regards to side effects.

Another user warned:

If you have BV, please ask for the gel. It is expensive, but this pill is so hard on your body.

Three OM reviewers discussed stopping the medication. One reviewer skipped multiple doses because she was “scared to take [OM] again” due to vaginal swelling and irritation. The remaining 2 reviewers considered stopping OM. One had an adverse reaction to alcohol:

I am tempted to stop at 7 [of 10 days of treatment], but I really want this infection gone at once.

Another reviewer said:

I only have 5 days left, but really debating on whether or not to go ask for something different…[because the side effects were] making me miserable!!

### Side Effects of the Reviewed Products

Among all three products, a portion of reviewers reported side effects. Specifically, 33 reviewers mentioned experiencing side effects while taking OM (vs 2 who reported none, 5 not mentioned), 27 for VM (vs 1 none, 12 not mentioned), and 25 for VC (vs 10 none, 5 not mentioned). OM reviewers described a wide variety of severe and plentiful side effects. Most OM reviewers experienced multiple side effects from flulike symptoms, gastrointestinal (GI) issues, nausea and vomiting, chest discomfort, mental health issues (depression, anxiety), bleeding, pain, hair loss, fatigue, brain fog, and, to a lesser extent, discharge, yeast infections, and changes in urine. Avoiding alcohol “was the hardest part” for many reviewers, especially daily drinkers who “love a glass of wine with dinner every night.”

Similar to OM, a majority of VM users also experienced side effects. Common VM side effects included discharge described as white, clumpy, or stringy, and rarely bloody, followed by cramps and burning, which ranged from mild to extreme in intensity. A minority of VC reviewers experienced flulike symptoms, mental health issues (anxiety, depression), brain fog, nausea, changes in urine, GI discomfort, and headaches; only 1 reviewer described an allergic reaction (VM and OM). Side effects from VM and alcohol use were mixed, with 1 reviewer saying they drank alcohol and did not experience vomiting. Only 2 VM reviewers experienced a severe yeast infection.

The most common side effects reported by VC reviewers were leakage/discharge, followed by yeast infection, burning/irritation, GI discomfort, itching, headaches, and fatigue. Severe and treatment-resistant yeast infections were most common in VC reviewers and the reason some VC reviewers “regret not choosing” a different BV product, such as metronidazole. Additionally, many of the reviewers who only experienced leakage/discharge felt this was typical of vaginal medications and were not bothered by this side effect, especially when their symptoms resolved.

### Characteristics of the Reviewed Products

Some reviews discussed characteristics of the products (attributes, taste, consistency, method of delivery, packaging, etc) that reviewers liked and did not like. An overwhelming number of OM reviewers complained about the product’s “horrible,” “nasty,” and “terrible” taste. Some OM reviewers reported that it dissolved so quickly in their mouth that they could not swallow it fast enough to avoid the taste. Overall, reviewers felt the vaginal products (ie, VC and VM) were easy to use. A few VM reviewers likened it to “a thin tampon,” and one said:

If you are used to using tampons, this will be very easy to apply.

Not all reviewers preferred vaginal medications. One VM reviewer who does not use tampons said she was “not too fond of inserting things inside of my vagina…” but that “the gel is so smooth it almost feels like nothing.” A few VC and VM reviewers felt the products have a pleasant cooling or smooth sensation. One VC reviewer said:

The only annoyance I had was that the suppository doesn’t stay in the vagina, and I had to push it back in and lie down until it dissolved.

Another VM reviewer said she “…could have done without the mess of the gel and the applicators, though”; however, 2 VM reviewers remarked that the “gel did not come out” after insertion, and 1 said she “even had sex one night after using and neither of us noticed the gel.” One VC reviewer liked the one-dose treatment.

### Cross-Product Discussions

A portion of VC, OM, and VM reviewers discussed using other products to treat BV. The largest group were reviewers who said they tried but did not find relief from OTC products—such as boric acid and probiotics—and sought a prescription medication. Many tried multiple prescription products. Another group of reviewers discussed taking an OTC product before, during, and after taking a prescription medication as a preventative. Finally, a minority of reviewers said they stopped taking a prescription product and only took OTC products. One VC reviewer said boric acid vaginal suppositories and once-daily acidophilus were “the best cure” because “it helps with pH and kills the odor.” Other OM users also tried tea tree oil, vitamin C ovules, and vaginal washes, which did not alleviate their infection. Several reviewers said their doctors recommended eating yogurt or taking OTC products (probiotics, boric acid), in addition to prescription treatment. It was more common that reviewers would switch to VM after OM due to its “horrible, horrible side effects like nausea, bad taste in mouth, and a yeast infection.” Another OM reviewer said:

If this is for BV, I urge you to try boric acid, probiotic, and the gel form rather than tablets. Alternatively, clindamycin can be considered as well.

Among VC reviewers who mentioned cross products, the majority preferred VC over metronidazole because it has fewer side effects; one reviewer said, “Please give it a try if you are fed up with metronidazole. This is a great alternative.”

### Seeking Information and Guidance

Many reviewers said they read “all the reviews” before taking a product, which helped them decide whether to take it. Reading reviews left most reviewers “scared” or “terrified” to take the product, but only a few said they refused to take a product because of what they read. Others said they were “really glad I did not read the reviews” before using it. Some reported delaying treatment until the infection worsened. When they finally took it, many said they were relieved when the side effects were mild and worried over nothing.

Reviewers also learned about side effects and how to minimize them from reading reviews. One reviewer said:

I wish my [doctor] would have warned that a yeast infection was possible.

Most OM reviews (n=24, 60%) focused on ways to minimize the “horrible”-tasting product.

Some used their reviews to ask questions. The most common question was how long after treatment should they abstain from having sex. A few reviewers had questions about whether their male partners should be tested or treated for BV:

I’m curious to know do my partner need to be tested before we have intercourse again? That wasn’t explained to me at my doctor visit.

Other reviewers turned to the reviews for guidance on treatment length, for example:

I was informed to complete a 10 day which is confusing because other reviews only state a 5-day term. Has anyone else been told to complete a 10 day?”

Several wondered how long they would experience side effects, such as a metallic taste or leakage/discharge. For example, 1 VC user said:

It’s a pasty stuff coming out...Is it the cream still coming out? How long does it take for it to totally come out?

### Airing Frustrations

#### State of the Science

Some reviewers expressed frustration about the state of BV treatments. One reviewer said:

I do not think the scientists work hard enough on a permanent BV solution. But at least, there are temporary fixes for now, I suppose.

Another reviewer said:

How could something that seems to be so simple of an issue that so many women experience, be treated with medication that causes so many horrible side effects!?

A third reviewer expressed a similar sentiment:

I think it is ridiculous that something that is so common in women is so hard to get rid of and this medication along with another one is the only thing that doctors can treat you with.

#### Doctor Interactions

Multiple reviewers disclosed interactions they had with their doctors. Some reviewers reported their doctors refusing to test or treat them for BV. One reviewer said:

When I got tested from my gynecologist, she told me that I was borderline BV...so they refused to give me anything.

Another reviewer said she was “switching to another OB gynecologist” because she was prescribed a product without being tested beforehand and suffered stomachaches, nausea, vomiting, and lethargy.

Others reported doctors encouraging them to complete treatment despite severe side effects. One reviewer appeared to have an allergic reaction to a product:

I went to my doctor because I was out of work because of this stuff. She had me finish the medicine.

Some reviewers complained that their doctor or pharmacist did not but should have warned them to avoid alcohol and alcohol-containing beauty products (eg, sprays, lotions) and reported suffering extreme nausea and vomiting. Another reviewer who stopped taking a product due to side effects said her “VERY annoyed” doctor did not believe it could cause diarrhea and “found it ‘hard to believe’ that the vaginal burning could be ‘that bad’.” When this reviewer asked for an alternative medication, her doctor “snapped that there ‘aren't any’ and told me to call my gynecologist.”

Some reviewers were annoyed that they were not offered alternative treatments with fewer side effects sooner. For example, one reviewer who was taking a “disgusting tast[ing]” product that made her feel “horrible” for 6 months reported she “finally went to a gynecologist that listened…he prescribed me [VM] instead of pill form.” Other reviewers reported having prolonged, repeat infections before their doctor switched medications. For example, one user said:

Apparently my body has become immune to [metronidazole], so my doctor wanted me to try

clindamycin

Others felt their doctor did not listen to their product preferences. For example, one reviewer said she asked her doctor “why he would not give me pills instead as I really did not want to insert anything into such an irritated area, but he insisted this was the best method.”

## Discussion

### Principal Findings

This mixed methods study examined patient-reported sentiment toward three of the most prescribed BV medications using data from online drug review forums. Overall, findings show that people want a BV treatment that is easy to use, quickly alleviates symptoms, and has minimal side effects. Although current BV treatment prescribing patterns [33] recommend OM as a first-line therapy, followed by VM and VC, this study found a preference for vaginal products overall. Additionally, VC had higher star ratings compared to VM and OM, which was bolstered by qualitative findings suggesting that VM and VC are effective and typically relieve symptoms 1-2 days faster than OM. Moreover, the side effects of VM and VC are usually local (eg, leakage/discharge, yeast infection, and vaginal irritation), whereas OM is associated with a broader range of systemic and more severe side effects, including adverse alcohol interactions well documented in the literature [34].

Honoring patient preferences—including the desired route of administration and concerns about side effects—is essential for improving BV treatment satisfaction and outcomes [35,36]. Health care providers should engage patients in discussions about lifestyle factors that may influence treatment choice, such as alcohol use [34], and BV triggers [37], such as sexual and hygiene practices [3,4] and the risk of reinfection from an untreated partner [38,39].

Findings from this study, along with previous research, show that patients will use internet searches to self-diagnose BV [17] and seek self-help remedies, such as OTC medications [40], even when this leads to misinformation [41]. This is unsurprising, given the negative psychosocial [42] and quality-of-life effects associated with BV [18,19]. For example, studies have shown that as many as 50% of BV patients mistakenly believe they have a yeast infection and take OTC antifungal treatments [43]. The remaining 50%, often those with recurrent BV who correctly identify their condition, frequently try OTC treatments, probiotics, or home remedies before seeking professional care [44].

Health care providers have a unique opportunity to educate patients about BV, dispel common myths, and inform them about available treatments. For instance, some OM reviewers expressed surprise and satisfaction upon learning that metronidazole is available in vaginal form. Others wanted their providers to proactively discuss side effects, particularly avoiding alcohol and alcohol-containing beauty products while on metronidazole. Patients also need information about when they resume sexual activity. Comprehensive education, whether provided in clinical encounters or through marketing and outreach efforts, is needed to help patients make informed decisions without reliance on online forums, where misinformation can be found [41]. At a broader level, public health campaigns can help reduce the stigma surrounding BV, overcome feelings of embarrassment associated with the disease, and empower patients with accurate information and support.

### Strengths and Limitations

Findings should be interpreted while considering the study’s strengths and limitations. Although the study relied on cross-sectional data, potentially from patients with a greater willingness to share their experiences via online platforms, it is a strength that the study used a large sample and the proportion of products reviewed largely mimics guideline-prescribing patterns. Another limitation is that the data are self-reported and assumed to be accurate, truthful, and from the person who used the product as directed, which cannot be verified. It is not known whether reviewers were paid money or given free products for their review. To submit a review, users must create an account, which is a strength as this may deter bots or fake reviews. Furthermore, each website uses its own security measures to limit fake/bot-generated reviews.

Another limitation of the study is that the NRC lexicon is not specific to BV, medical conditions, or pharmaceutical treatments. Therefore, BV-specific terms may not be captured in these analyses. Given the exploratory nature of this study, future studies should consider using other internet sources where patients provide robust information about their health care needs, symptoms, experiences, and recommendations. Combining data from several websites could be used to validate or create a lexicon that is more specific to a given medical condition.

A strength of the study is that it leveraged both qualitative and quantitative data to include patient-reported ratings and SA scores. Including a traditional qualitative analysis may have helped overcome critiques about the accuracy of SA approaches, which depend on the lexicon used, how well it matches subjective interpretation, and complex speech patterns (sarcasm, irony, negation, misspellings, etc). At multiple points, we used a human assessment of reviews to increase rigor and validity. As is the case with all qualitative studies, the sample is not representative of the population, and we only analyzed a subset of the 40 longest reviews per product. Although only one author (AC) performed the qualitative analysis, a strength is that the themes aligned with the double-coded and verified classifications. Because a reviewer could write anything they wanted, caution should be used when interpreting counts in the qualitative results, as some only discussed their experience with BV, not information about the reviewed product. Although we selected the 40 longest reviews for each product for the qualitative analysis to focus on the richest data, these reviews may have been different (eg, represent more extreme experiences) than an average review. Due to the study design, we were unable to engage with the review to ask follow-up or clarifying questions.

### Conclusion

Despite being the most common cause of vaginal discharge in people of childbearing age in the United States, innovation toward treatment options for BV are limited. This study’s findings substantiate the need for a BV treatment that that is easy to use, quickly alleviates symptoms, and has minimal side effects. Considering how online resources are a go-to source for patient health information, these findings also underscore the need for comprehensive education to help patients differentiate facts from myths related to BV disease. Finally, considering patient preferences when prescribing BV treatment options is important for achieving treatment success and ensuring satisfaction.

## Appendix

Multimedia Appendix 1 Positive emotion word proportions by product.

Multimedia Appendix 2 Negative emotion word proportions by product.

## Abbreviations

AE: adverse event
BV: bacterial vaginosis
FDA: Food and Drug Administration
GI: gastrointestinal
NRC: National Research Council Canada
OM: oral metronidazole
OTC: over the counter
SA: sentiment analysis
VC: vaginal clindamycin
VM: vaginal metronidazole
