# Supplementary figures and images for: Understanding Patient Perceptions of Bacterial Vaginosis Treatments: Mixed Methods Sentiment Analysis Study of Online Drug Review Forums
Source: Online J Public Health Inform. 2025 Oct 10;17:e71720. doi: 10.2196/71720 (PMC12552819; doi:10.2196/71720)

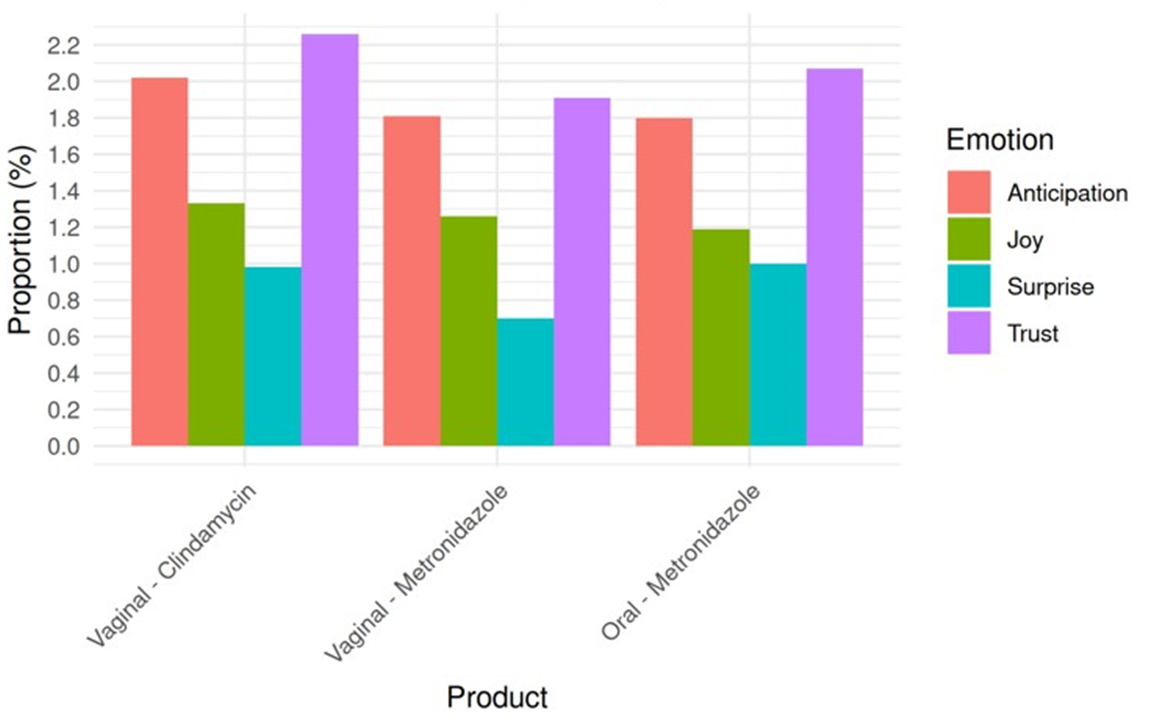

Supplement: Multimedia Appendix 1 [file ojphi_v17i1e71720_app1.png]

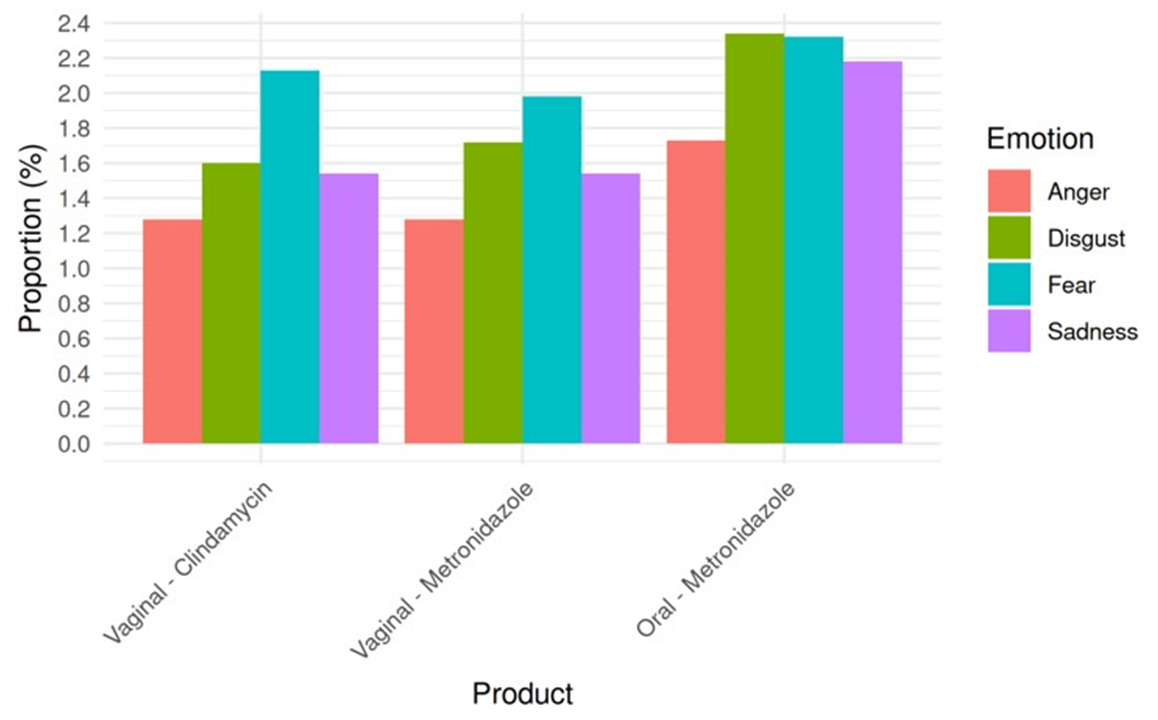

Supplement: Multimedia Appendix 2 [file ojphi_v17i1e71720_app2.png]
